# Reducing Alcohol Misuse and Promoting Treatment Initiation Among Veterans Through a Brief Internet-Based Intervention: Protocol for a Randomized Controlled Trial

**DOI:** 10.2196/59993

**Published:** 2024-08-22

**Authors:** Eric R Pedersen, Jordan P Davis, Justin F Hummer, Kathryn Bouskill, Keegan D Buch, Ireland M Shute, Reagan E Fitzke, Denise D Tran, Clayton Neighbors, Shaddy Saba

**Affiliations:** 1 Department of Psychiatry and Behavioral Sciences Keck School of Medicine University of Southern California Los Angeles, CA United States; 2 RAND Santa Monica, CA United States; 3 Department of Psychology University of Colorado Colorado Springs Colorado Springs, CO United States; 4 Department of Psychology University of Houston Houston, TX United States; 5 Dworak-Peck School of Social Work University of Southern California Los Angeles, CA United States

**Keywords:** military, mental health, substance use, mobile, PTSD, posttraumatic stress disorder, drinking, mobile intervention, digital intervention, alcohol, alcohol misuse, veterans, young adults, depression, alcohol use, veteran health, veteran

## Abstract

**Background:**

Young adult veterans who served after the September 11 attacks on the United States in 2001 (ie, post-9/11) are at heightened risk for experiencing behavioral health distress and disorders including hazardous drinking, posttraumatic stress disorder, and depression. These veterans often face significant barriers to behavioral health treatment, and reaching them through brief mobile phone–based interventions may help reduce drinking and promote treatment engagement.

**Objective:**

Following a successful pilot study, this randomized controlled trial (RCT) aims to further test the efficacy of a brief (ie, single session) mobile phone–delivered personalized normative feedback intervention enhanced with content to promote treatment engagement.

**Methods:**

We will conduct an RCT with 800 post-9/11 young adult veterans (aged 18 to 40 years) with potentially hazardous drinking and who have not recently received treatment for any behavioral health problems. Participants will be randomly assigned to the personalized intervention or a control condition with resources for seeking care. The personalized normative feedback module in the intervention focuses on the correction of misperceived norms of peer alcohol use and uses empirically informed approaches to increase motivation to address alcohol use and co-occurring behavioral health problems. Past 30-day drinking, alcohol-related consequences, and treatment-seeking behaviors will be assessed at baseline and 3, 6, 9, and 12 months post intervention. Sex, barriers to care, posttraumatic stress disorder, depression, and severity of alcohol use disorder symptoms will be explored as potential moderators of outcomes.

**Results:**

We expect recruitment to be completed within 6 months, with data collection taking 12 months for each enrolled participant. Analyses will begin within 3 months of the final data collection point (ie, 12 months follow-up).

**Conclusions:**

This RCT will evaluate the efficacy of a novel intervention for non–treatment-seeking veterans who struggle with hazardous drinking and possible co-occurring behavioral health problems. This intervention has the potential to improve veteran health outcomes and overcome significant barriers to treatment.

**Trial Registration:**

ClinicalTrials.gov NCT04244461; https://clinicaltrials.gov/study/NCT04244461

**International Registered Report Identifier (IRRID):**

DERR1-10.2196/59993

## Introduction

### Background

Americans who served in the US military during or after September 11, 2001, and have since been discharged (post-9/11 veterans) experience concerning rates of mental health disorders, with an estimated prevalence of depression and posttraumatic stress disorder (PTSD) ranging from 10% to 30%, respectively [1-5], and often co-occurring [6]. Alcohol use disorder (AUD) is the most common substance use disorder among veterans [7-10] and it, too, often co-occurs among veterans with depression or PTSD [11-14]. Approximately 1 in 10 post-9/11 veterans who are seeking any care within the Veterans Health Administration meet the criteria for an AUD [9], and studies show that upwards of 40% of these veterans drink alcohol at levels that place them at risk for negative alcohol-related consequences (ie, hazardous drinking) [10,15-18]. Veterans younger than 40 years old drink more heavily than older veterans [9,10] and may be at heightened risk for developing an AUD later in life, making early intervention and prevention efforts for younger adult veterans essential.

Despite the availability of evidence-based behavioral health care (ie, care for mental health or substance use disorders) through Veterans Affairs (VA) and in private care settings [19-22], not all veterans with behavioral health needs use these services. Estimates suggest that over 50% of post-9/11 veterans meet screening criteria for a mental health disorder (including AUD), but less than half receive care [23-29]. Those who do often receive inadequate care to address their needs, such as seeing a mental health provider only once or twice for an intake without follow-up or not completing a doctor’s full planned prescription for psychotropic medications [30-32]. Veterans who engage in heavy drinking and have a co-occurring PTSD and depression diagnosis have particularly difficult barriers to seeking care [33]. It is important to target heavy alcohol use early after discharge and in young adulthood so both alcohol use and co-occurring mental health problems do not become chronic or more difficult to treat.

Veterans may not receive needed care for a variety of reasons. Barriers to care include perceived stigma from others (eg, a belief that colleagues, family, and friends would respect them less), fear of repercussions or career harm (eg, concerns that their career would not progress if they seek treatment), beliefs that their drinking behavior is normative among other veterans and not in need of change, lack of awareness of treatment options, concerns about high costs, limited access due to residency in rural areas, and beliefs that they can handle their problems on their own or that available treatments are not effective [27,29,34-38]. Women and racial or ethnic minority veterans may have additional barriers related to childcare responsibilities, apprehension about using VA facilities, discomfort and mistrust of providers, and discrimination within health care systems [39].

Internet and mobile phone–based treatment approaches represent a potentially important avenue through which to help veterans overcome barriers to accessing the care they may not otherwise pursue*.* Existing outreach efforts target veterans already seeking services at the VA (eg, care for physical injuries) through public media campaigns and announcements in primary care clinics. However, these efforts do little for the largely neglected population of veterans who have not received care from the VA or for those who may prefer to seek care elsewhere but are either unaware of or disinterested in their options [33,40]. In addition, stand-alone internet-based alcohol interventions for veterans are lengthy and characterized by high attrition [41-45].

In an effort to reach veterans outside of VA settings, we designed and pilot-tested a single-session intervention [46] for a cohort of young adult veterans (ie, aged 18 to 34 years) who were recruited on social media websites. In the intervention, which targeted veteran drinkers who were not necessarily seeking treatment for alcohol-related concerns, veteran participants were shown normative data of their sex-specific veteran peers (ie, other male veterans and other female veterans) [47,48] to correct their overestimations of peer veteran drinking behavior and to provide a comparative reference for their drinking behavior (ie, to demonstrate that their veteran peers likely drink less than an individual may think other veterans drink, or less than they, in fact, do). In this pilot randomized controlled trial (RCT; ClinicalTrials.gov NCT02187887), we found small but significant intervention effects favoring the intervention 1 month post intervention on all prespecified drinking outcomes: past 30-day drinks per week (*d*=0.47), average number of drinks per occasion (*d*=0.17), binge drinking days (defined at 5 drinks in a row for males and 4 drinks in a row for females; *d*=0.18), and alcohol-related consequences (*d*=0.17) compared to control [49].

As concluded in a recent review of brief internet-based interventions for young adult alcohol misuse [50], from a public health perspective, being able to reach a large number of individuals and provide them with interventions that lead to even modest effects is beneficial at a societal level. Such modest effects from a single-session program may be important as an individual begins to evaluate one’s personal alcohol use and considers making a change, which may include seeking out formal assessment and more intensive treatment. The personalized normative feedback (PNF) approach offers a reference point for an individual’s personal drinking habits compared to peers, and similar to a checkup model [41,51], allows one to consider making a change to their drinking. Though PNF interventions are efficacious, these effects are often small and short-lived [52]. Thus, there is room for PNF interventions to expand beyond short-term effects on drinking and be used as a vehicle to connect those who have never received an intervention before with more intensive services. For veterans engaging in heavier problem drinking, sustained changes in drinking may be better obtained through in-person individual or group interventions, as opposed to a one-time brief intervention. For veterans who use alcohol to cope with or manage PTSD or depression symptoms, more intensive co-occurring disorder treatment may be indicated, whereby they can learn more adaptive techniques to cope with negative affect. In our pilot study, we found that almost twice as many PNF condition participants as control participants reported increases in motivation and likelihood to seek AUD treatment simply after viewing the PNF [53], alluding to the promise of the approach in targeting treatment initiation among the group of veterans who have not engaged in care or have had substantial barriers or challenges doing so. If the brief intervention prompted veterans to think more about making a change to their drinking, it may be a tool to reach those who may not have otherwise considered change and to connect them with services.

### This Study

To build upon the pilot trial, we designed this study to test the longer-term effects (ie, 12-month outcomes) of a brief PNF intervention on drinking and treatment (ie, motivation and preparation for; actual initiation) outcomes. For this larger study, we expand upon the original pilot intervention content, which primarily focused on correcting misperceived norms of veteran peer alcohol use, to include empirically informed approaches to increase motivation to address one’s alcohol use and co-occurring behavioral mental health problems (eg, depression, anxiety, and PTSD) in more formal care settings (ie, those beyond a one-time brief intervention such as care with a provider at a VA). In doing so, we will evaluate intervention effects on readiness to initiate behavioral health care, preparatory behaviors toward seeking that care, and any actual care initiation in the postintervention period. To evaluate the impact of the intervention on these outcomes, we intend to enroll and randomize 800 participants into either the PNF intervention (n=400) or a resources-only control condition focused on providing information related to care services (n=400). In the protocol that follows, we describe our recruitment methods and target sample, list our measures for outcomes and moderators of intervention effects, discuss the intervention content in detail, and lay out the proposed analytic plan to examine the efficacy of the brief internet-based intervention on alcohol use and treatment initiation outcomes.

## Methods

### Trial Design Overview

**Figure 1:**
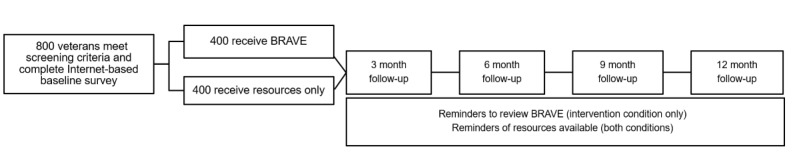
Flow of proposed randomized controlled trial. BRAVE: Building Resilience and Agency through Veteran Engagement.

This RCT (ClinicalTrials.gov NCT04244461) was designed to test the efficacy of a single-session brief mobile phone–based intervention with young adult veterans. The intervention will be a mix of narrated video, audio, text, and graphics that contain personalized content emphasizing the veteran’s values, their goals for treatment initiation, and resources to help them connect with behavioral health care. Veteran participants aged 18 to 40 years will complete a baseline survey, be randomized to the intervention or a control condition, and then complete four follow-up surveys at 3, 6, 9, and 12 months post baseline. At each follow-up period, all participants will be emailed a list of resources to help connect them to behavioral health services, as well as a link to the intervention website after each follow-up assessment. The intervention is named Project BRAVE (Building Resilience and Agency through Veteran Engagement), with an accompanying logo, to help veterans recognize the study during recruitment and reminder correspondence (Figure 1).

### Specific Aims and Hypotheses

For the first aim, we intend to conduct an RCT of the BRAVE intervention by randomly assigning veterans with potentially hazardous drinking patterns, but who have not received recent treatment for drinking or any other behavioral health problem, to receive either the BRAVE intervention or a resources-only control condition. We hypothesize that BRAVE participants will drink less (fewer days per week, fewer drinks per occasion, and fewer binge drinking days), experience fewer negative alcohol-related consequences, and report more treatment readiness, preparatory behaviors, and initiation behaviors at 3, 6, 9, and 12 months post intervention, relative to control participants. For the second aim, we will determine for which subgroups the intervention works best. We hypothesize BRAVE participants who report more baseline barriers to care (eg, perceived public stigma beliefs that others would view them negatively for seeking care, not having time due to work or family responsibilities), baseline high levels of PTSD or depression symptoms, and higher baseline problematic alcohol use will benefit most from the intervention in terms of drinking reductions and treatment initiation outcomes. Sex (male and female) will be examined as an exploratory moderator, which expands on veteran work that traditionally examines male veterans only, which is important considering unique female-specific factors that affect treatment engagement [39].

### Participant Recruitment and Eligibility

The BRAVE intervention is designed for mobile phones with internet capabilities and is targeted toward young adult veterans. Though the intervention is designed for use on mobile phones, it can be accessed through computers and tablets. Given the focus on a younger veteran cohort, eligibility criteria include (1) US veteran who has been discharged or separated from the Air Force, Army, Navy, Marine Corps, or Coast Guard (participants could have also been in the reserves or guard units, but not on active duty), (2) between the ages of 18 and 40 years, (3) a score on the 10-item Alcohol Use Disorders Identification Test (AUDIT [54]) of 4 or greater (men) or 3 or greater (women), which represents cutoff scores to identify those who may benefit from interventions to reduce alcohol misuse, (4) no upcoming appointments at the VA or elsewhere to receive treatment for a behavioral health concern (such as depression, PTSD, or alcohol use concerns), and (5) no appointments in the past six months for behavioral health treatment at a VA or elsewhere. The purpose of these latter 2 criteria is to reach participants outside of traditional care settings (ie, those who may need care but who are not receiving it) and to measure the effect of the intervention on treatment initiation. Participants also need access to the internet via a computer, tablet, or phone; a working email address; and the ability to read and understand English. Based on these criteria, we will recruit 800 participants who will be randomly assigned to the BRAVE intervention (n=400) or a resources-only control condition (n=400) that presents resources for care seeking (eg, location of local VA and links for connecting with local veteran service organizations) without any personalized content.

### Procedures

Participants will be recruited with the aid of a recruitment platform, BuildClinical, which uses machine learning and data mining procedures to target internet ads to recruit the sample population from a variety of general internet and social media sources (eg, Instagram, Facebook, Google, and health websites). BuildClinical uses targeted ad imagery and text to ensure we meet the recruitment needs of the study, including aiming for 10%-15% women veterans to match the growing number of post-9/11 women veterans. The pilot intervention study successfully enrolled 18% of women veterans [49]. Participants will click on ads displayed and be directed to a website with a description of the study. If interested, they can fill out a web-based screening form to assess eligibility and answer questions to help determine that they are not falsifying responses to gain access to the offered incentives. We have developed procedures to identify and exclude nonveterans from enrollment, which includes asking about first-hand knowledge through questions that must be consistent across responses (eg, branch, rank, pay grade, and job in the military must all make sense when considered together) and ensuring responses are identical when asked variations of the same question at different times throughout the survey. Once participants pass through the initial validation checks, we will call each potential participant to verify the information they provided (eg, ensuring phone responses match survey responses to items such as branch and rank). If the individual’s responses are deemed credible and they remain interested in participation, the individual will be enrolled as a participant following a discussion of the study procedures and consent document. Once enrolled, participants will fill out a baseline survey and then be randomized to one of two conditions: the BRAVE intervention or a resources-only control condition.

### BRAVE Intervention Condition

**Figure 2:**
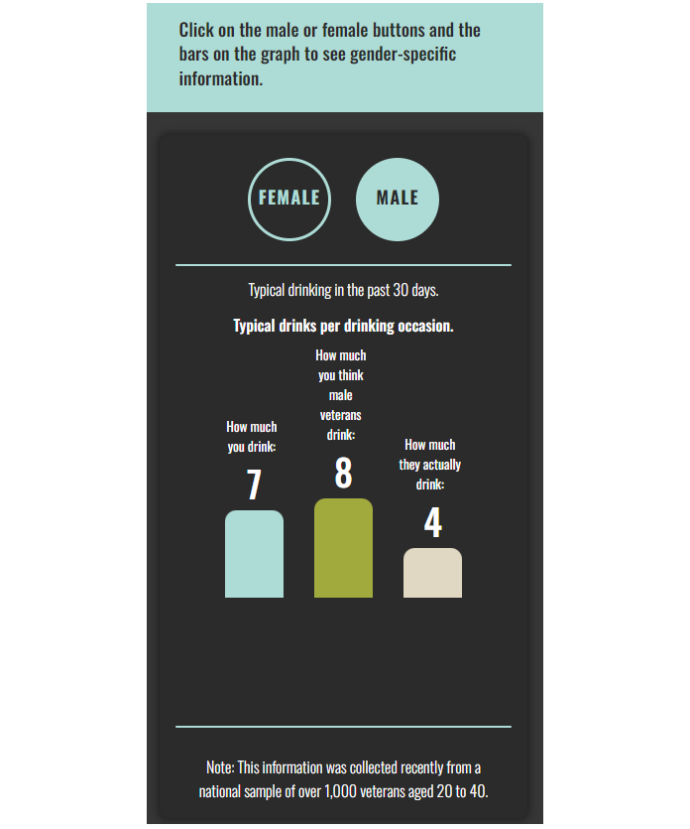
Sample of drinking personalized normative feedback.

The intervention was designed to build upon the piloted PNF intervention with veterans from our prior work [49]. Similar to other young adult groups, veterans misperceive the drinking behavior of their peers and these misperceptions are associated with their drinking behavior [48]. PNF is one of the most frequently used efficacious drinking interventions for young adults [52] and we have shown promise in the stand-alone approach for young veterans [49]. In the PNF, individuals are presented with normative data that challenges their beliefs that similar peers drink as heavily as the individual perceives they do. Using graphics and descriptions of norms theory (eg, we pay attention to patterns outside the norm and come to believe those deviations are more normative than they are), individuals can see that their perceptions about others may be incorrect and that they may be drinking more than their peers. In BRAVE, participants are presented with PNF approximately midway through the larger, multicomponent intervention. First, participants are asked about their drinking behavior and the behavior of their peers in the past 30 days. Participants indicate how many drinks they consumed on a typical drinking day and how many drinks they think other male and female veterans drank on a typical drinking day. They then indicate how many days in the past 30 days they drank 4 (for females) or 5 (for males) drinks on an occasion (ie, binge drinking) and how many days they think male and female veterans drank these amounts. PNF is then displayed for both male and female drinking norms to all participants so veterans can see that heavy drinking is not the norm among both sexes (see example in Figure 2). Norms for drinking were derived from our prior work with 1230 veterans [55].

We expanded the PNF intervention to include content designed to encourage veterans who were not currently seeking care for alcohol or mental health concerns to more strongly consider and actually seek care. The intervention includes a mix of videos, audio, text, graphics, and text-based components. There are 2 veteran peers as narrators (one male and one female) who walk veterans through a 20- to 30-minute personalized intervention. Veteran peers were selected for the program given prior work showing the importance of peers in helping change attitudes and behaviors in health interventions [56], particularly among veterans with mental health and substance use concerns [57,58].

BRAVE begins with the clarification of personal values, with the selection of values and related content that highlights the ways in which veterans live according to those values. Veterans are given a set of 16 values and asked to select which resonates with them. Examples of values presented include Honor (“To adhere to what is right with great respect”), Self-control (“To be disciplined in my own actions”), Courage (“To be brave and strong in the face of fear, threat, or difficulty”), and Empathy (“To try and understand the perspective of others and what they are feeling”). They are also given the option to indicate their own values not included in the sample list. This component is based on the “values card sort” exercise originally created as a tool in Motivational Interviewing [59], and used heavily in Acceptance and Commitment Therapy [60]. The purpose of using these intervention components is to help veterans identify and reflect on what is most meaningful and important to them in their lives; this may include seeking care for any mental health concerns they may have. Clarifying values will then help a veteran move forward on important committed actions, defined as actions in the direction of what someone cares about even in the presence of obstacles (eg, addressing problematic alcohol use or seeking care) [61]. Values clarification is followed by content to help veterans overcome identified barriers to care, such as challenging unhelpful beliefs about treatment (eg, “I can handle my problems on my own,” “Providers will force me to take medication,” and “My symptoms are not severe enough to deserve care”) by providing information about what to expect when seeking care, with practical resources for where to seek help. Four main topic areas are presented to veterans, where they can click to learn more about each area: “What is mental health treatment like?” “What is the format of mental health treatment?” “How much of a commitment is mental health treatment?” and “Where can I get mental health treatment?” PNF is reviewed for alcohol use as described above, followed by normative feedback that helps to challenge the perceived public stigma that many veterans have about seeking care [62]. For example, the narrators describe studies that show that other veterans would not see someone as weak for seeking care; in fact, they would encourage those who need help to get the help they need. Preliminary work shows the promise of showing individuals information that corrects their misperceptions about perceived public stigma (eg, informing them that most people think that having a mental health problem is nothing to be embarrassed about [63]). Within the intervention, veterans are prompted to identify their personal values; these values are repeatedly referred to throughout the content of the intervention, including when veterans make goals for themselves about seeking care or making a positive change in their lives (eg, the intervention prompts them to mindfully consider what changes they can make to live more in accordance with their values [64]). Veterans are asked about some reasons for seeking behavioral health care that resonates with them, such as “My family life may improve,” “I may gain personal insight and awareness,” and “I may feel more motivated to do the things I want to do.” These reasons for seeking care are presented alongside their values and veterans are asked to consider if seeking care would help them live more in line with their values. Informed by Cognitive Behavioral Therapy [65] and Motivational Interviewing [51,66] theory and practice, content is presented in a nonjudgmental way, with veterans able to make their own choices about making a change to their drinking or pursuit of behavioral health care.

In the penultimate section of the program, veterans are asked to make immediate, short-term, and long-term goals, with encouragement from the narrators to work toward those goals in the next few months [67]. Veterans type in their goals related to any area of their life where they want to make a change (eg, “I want to drink less,” “I want to address my anxiety and reduce stress,” and “I want to improve my relationship with my kids”) and are asked to make sure these goals align with their values. Prompts for immediate (ie, something small and simple that they can easily accomplish within the next 24 hours), short-term (ie, things they can do in the service of their values-based goal within the next few days and weeks), and long-term (ie, actions that will move them closer to their values-based goal over the next few months and years) goals are provided.

The intervention concludes with a summary of the content reviewed, as well as personalized resources, including where to get help (eg, the location of nearest care centers based on the participant’s ZIP code and crisis assistance) and information about local peer support groups and veterans service organizations. In the RCT, participants will receive a login for the intervention and can return to it at any time during the study. At each follow-up survey, they will be sent the link to the intervention with their login information and encouraged to review the content again.

### Control Condition

Participants assigned to the control condition will receive a link to a website with resources similar to those provided at the end of the BRAVE intervention. On the site, veterans are able to find care centers, peer support groups, and veterans service organizations by clicking through the organized content. Participants will be asked to explore the content on the website for about 20 to 30 minutes, to match the length of the BRAVE intervention. They can return to the website at any time. After each follow-up survey, the control participants will be sent the link to these resources again to have it easily accessible.

### Measures

#### Overview

Measures will be collected at screening, baseline, and all follow-up time points through confidential web-based surveys. After completing the screening survey and becoming enrolled, participants will receive a US $20 gift card for each survey they complete (a total of US $100 for all surveys).

#### Demographics and Background

Participants will fill out several demographic items that will be used to describe the sample. These include age, sex at birth, gender, race, ethnicity, income level, military branch, military rank and pay grade at discharge, number and location of deployments, education level, marital status, and number of children. Participants will also complete a measure of combat exposure to assess for presence and severity of combat experiences while in the military [27].

#### Primary Outcomes

Primary outcomes are drinking behavior (past 30-day frequency, quantity, and number of binge-drinking days), negative alcohol-related consequences, and treatment initiation. Three past 30-day drinking outcomes will be assessed through single-item self-reports: drinking days, average drinks per occasion, and binge drinking days. Alcohol-related consequences in the past 30 days will be assessed with the Short Inventory of Problems [68], which is modified from the Drinker Inventory of Consequences [69]. The treatment outcome will be measured as treatment initiation in the time period after the intervention (ie, post baseline). Treatment initiation is assessed with an item asking participants to indicate if they have seen a provider at a VA or non-VA care setting for therapy, counseling, support groups, hospitalization, or medications for any concerns related to alcohol or substance use (eg, if they sought help to drink less, or attended groups to stay abstinent from cannabis or other drugs) or mental health concerns (eg, to help with stress, anxiety, depression, nightmares) in the past 6 months (at baseline) and past 3 months (follow-ups). Providers are defined for participants as a mental health or behavioral health provider (eg, psychiatrist, psychologist, social worker, mental or behavioral health nurse, or other providers), a general medical provider (eg, primary care doctor, physician assistant, and nurse practitioner), or an addiction specialist (eg, addiction counselor). Care is specified as either in-person or via telehealth.

#### Secondary Outcomes

The decision to seek care and then actual receipt of care can be a lengthy process. Given that participants may not receive any care during the 12 months of follow-up, we will also assess their readiness to receive care in the future and if they made preparations to receive that care. Preparatory behaviors will be specified as 6 behaviors participants would definitely do, probably do, probably not do, or definitely not do in the next 30 days to prepare for treatment (ie, looking at options on the internet; discussing with a partner, family member, or friend the options available; talking with a partner, family member, or friend about their experiences with treatment; contacting a provider to learn more about the options; contacting the insurance provider to see what options are available; and contacting a provider to make an appointment). Two readiness rulers will also assess participants’ readiness to seek care in the next 30 days in order to reduce their drinking and to address any mental health concerns they have from 0 (not ready at all) to 10 (absolutely ready).

#### Moderators

To examine the characteristics of participants for whom the intervention is most efficacious, we will include measures at baseline of perceived barriers to treatment, PTSD symptoms, depression symptoms, and AUD severity. Perceived barriers are assessed with the 15-item RAND Barriers to Mental Health Care item bank [70]. Items are rated on a scale from 1 (not at all) to 5 (very much) and reflect barriers of perceived stigma (eg, seeking care would be viewed as a sign of personal failure, others would lose respect for the person, and other veterans would see them as weak), fears about confidentiality (eg, people might find out about it), beliefs that treatment does not work, and difficulties finding time for treatment due to other responsibilities (eg, because of work or family). These items can be summed to receive a baseline severity of barriers score. PTSD symptoms will be assessed with the 20-item PTSD Checklist (PCL-5), a widely used measure for military and veteran populations with adequate reliability and validity for young adult military samples [71]. Depression symptoms will be assessed with the Patient Health Questionnaire 8-item (PHQ-8), a reliable and valid measure of depression used in research and practice for military and veterans [72-74]. The severity of AUD will be assessed with the AUDIT [54], a 10-item alcohol screen that identifies individuals who had probable AUD in the past year. The PCL-5, PHQ-8, and AUDIT can each be summed to create baseline symptom severity scores for each.

### Analytic Plan

#### Overview

Prior to the main aims analyses, we will assess for multivariate normality. The maximum likelihood estimator will be used if data are approximately normal and robust maximum likelihood will be used if the data are not multivariate normal. Missing data will be assumed to be missing at random and handled using direct maximum likelihood techniques. We will assess for differences in baseline demographics and military characteristics between the intervention (BRAVE) and control conditions. Any characteristic significantly different between groups will be controlled for in analyses.

To assess the effects of the intervention on continuous primary outcomes (drinking days, average drinks per occasion, binge drinking days, negative alcohol-related consequences, and treatment initiation) and secondary outcomes (preparatory behaviors, readiness to seek care ruler for alcohol, readiness to seek care ruler for mental health), a taxonomy of latent growth models will be estimated [75]. The primary outcome time point for this trial will be the baseline to first follow-up (ie, 3 months post baseline); however, we are interested in the past 30-day changes from baseline to each of the 3 additional follow-up timepoints (ie, baseline to 6, 9, and 12 months post baseline). To assess between-group differences in the intercept and growth factors, we will regress each on a dummy variable representing the intervention (BRAVE=1) and control (control=0) groups. For any count variables, negative binomial or Poisson distributions will be explored for better model fit. To understand the change in treatment initiation (binary outcome), we will use latent growth models with categorical outcomes, as well as estimate simple logistic regression at each time point. To understand the practical significance, we will calculate standardized mean differences (Cohen *d*) at all 4 follow-up time points for both between group and within group.

We will test for significant effect moderation by the following variables: barriers to care, PTSD, depression, and severity of AUD symptoms. To explore moderation, we will use simple linear regression models for primary outcomes at each time point. We will include an interaction term between each potential moderator and the intervention indicator (BRAVE vs control). Significant interactions between the intervention group and these moderators will be plotted with appropriate values and interpreted. Simple slopes will be calculated for all significant interactions. Given our prior work and the large sample size, we expect to have adequate numbers of participants in each cell for moderation analyses and will use established cutoff scores on the PCL-5 (score 33 or higher vs under 33), PHQ-8 (scores 10 or higher vs under 10), and the AUDIT (scores 8 or higher vs under 8). We hypothesize that those with greater barriers, PTSD, depression, and severity of AUD symptoms will benefit most from the intervention. Sex (male or female) will be included as an exploratory moderator.

#### Power Analysis

Given this study design (and standard *P*<.05; 2-tailed testing), we will have 80% power to detect small treatment effects between the groups; specifically, effects of d=0.22 at 3- and 6-month follow-ups and d=0.23 at 9- and 12-month follow-ups. For reference, our observed intervention effect sizes at 1 month post intervention in the pilot study were *d*=0.17 for average drinks, *d*=0.18 for binge days per month, and *d*=0.19 for consequences [49]. We have similarly found small effect sizes over 2 years of follow-up in other large-scale PNF studies [76-78]. We expect effect sizes to be small, though slightly larger than those in our prior work given we include a sample screening positive for hazardous alcohol use that could benefit from treatment, whereas prior work targeted general college students and young veterans drinking at less severe levels. As noted, given that this intervention targets those who may never have received care otherwise, small effects seen in brief interventions, such as this, are potentially clinically significant.

Our prior work evaluating treatment-seeking behaviors has found rates of preparatory and actual treatment-seeking by service members to increase by double following brief phone-based feedback [79]. Other work has similarly found rates of substance use treatment initiation to increase to upwards of 50% following in-person feedback–based approaches [80]; thus, we hypothesize that upwards of 50% of participants who receive the enhanced PNF will report actual treatment initiation at some point during the 12-month follow-up period. We will examine preparatory behaviors (ie, steps along the way to actual initiation) and motivation to seek care.

### Ethical Considerations

The internal review board at the University of Southern California approved all procedures for this study (HS-19-00532). All participants will review and sign an informed consent form prior to participation. Participants “opt-in” for this study and can remove themselves from participation at any time. Incentives for participation are specified above and will be made clear to participants at the time of recruitment. All collected data are confidential.

## Results

Data on primary and secondary outcomes will be collected as specified in the Methods section. We expect recruitment to be completed within 6 months, with data collection taking 12 months for each enrolled participant. Analyses will be conducted as specified in the Analytic Plan section above and begin within 3 months of the final data collection point (ie, 12-month follow-up).

## Discussion

### Principal Findings

The brief intervention we designed is intended to reach younger veterans who may have never otherwise considered seeking care for alcohol misuse or co-occurring mental health problems. BRAVE was designed to expand upon our prior pilot intervention study [46] that showed a PNF intervention yielded small intervention effects 1 month post intervention [49]. Given the ability of the internet recruitment method to access this difficult-to-reach population, combined with the intervention’s ability to prevent alcohol misuse and consequences among a population with high rates of co-occurring mental health symptoms, we included values clarification, goal setting, and content to address barriers to care to expand on the theoretical and empirical basis of PNF interventions, including theories of normative influence [81,82] and aspects of Motivational interviewing [66], to encourage veterans to consider making changes to their drinking behaviors early, either by reducing on their own or seeking formal treatment, before their alcohol use transitions to a more problematic AUD.

Should BRAVE demonstrate efficacy in reducing drinking and improving treatment engagement among veteran participants, there is the potential to reach large numbers of veterans given the ubiquity of mobile technology [83] and the potentially low cost and burden of accessing mobile health interventions [84]. There may be challenges associated with disseminating such an intervention more broadly, given that the target population involves veterans not engaged in treatment who therefore are not in contact with those who would traditionally recommend such an intervention (ie, care systems and providers).

Moderation analyses of this study could allow for the further tailoring of our treatment approach and better targeting of specific subgroups of veterans. That is, should our analyses reveal that veterans with specific clinical or demographic characteristics are more likely to evidence reductions in drinking and treatment-seeking behaviors, this could justify targeted dissemination of PNF interventions, like ours, to such veterans. On the other hand, should a subgroup of veterans be less responsive to BRAVE, researchers could consider further adapting such interventions to better target relevant symptom processes or demographic groups. Hypothetically, such an intervention could be tailored to better meet the needs of female or male veterans or could include modules specific to PTSD or depression symptomology.

### Limitations and Considerations

As the sample will involve relatively younger post-9/11 US veterans, it is unclear whether results will generalize to broader veteran samples, and in particular, veterans who are less comfortable with technology may have challenges engaging with an intervention such as BRAVE. Additionally, should the intervention demonstrate efficacy for reducing drinking and treatment-related outcomes, it will not be possible to disentangle whether it was the intervention as a whole or specific aspects of the intervention that were efficacious. Further dismantling studies of BRAVE may be necessary.

### Conclusions

The BRAVE intervention has much potential to provide intervention to a traditionally difficult-to-reach population. It leverages mobile technology and combines several intervention strategies to fit the needs of the specific clinical presentation and needs of the population. While we anticipate BRAVE to be efficacious, reaching veterans outside of research studies will be an essential next step for our team and for intervention developers more broadly. Dissemination and implementation of evidence-based mobile and internet-based interventions is a key component of continuing to improve behavioral health care access for veterans and other groups in need.

## Abbreviations

AUD: alcohol use disorder
AUDIT: Alcohol Use Disorders Identification Test
BRAVE: Building Resilience and Agency through Veteran Engagement
PCL: posttraumatic stress disorder checklist
PHQ-8: Patient Health Questionnaire 8-item
PNF: personalized normative feedback
PTSD: posttraumatic stress disorder
RCT: randomized controlled trial
VA: Veterans Affairs

## Data Availability

The data sets generated during or analyzed during this study are available from the corresponding author (ERP) upon reasonable request.
